# Prognostic and Therapeutic Role of Angiogenic Microenvironment in Thyroid Cancer

**DOI:** 10.3390/cancers13112775

**Published:** 2021-06-03

**Authors:** Assunta Melaccio, Lucia Ilaria Sgaramella, Alessandro Pasculli, Giovanna Di Meo, Angela Gurrado, Francesco Paolo Prete, Angelo Vacca, Roberto Ria, Mario Testini

**Affiliations:** 1 Operative Unit of Internal Medicine “G. Baccelli”, Department of Biomedical Sciences and Human Oncology, University of Bari “Aldo Moro” Medical School, 70124 Bari, Italy; assunta.melaccio@uniba.it (A.M.); angelo.vacca@uniba.it (A.V.); roberto.ria@uniba.it (R.R.); 2 Academic General Surgery Unit “V. Bonomo”, Department of Biomedical Sciences and Human Oncology, University of Bari “Aldo Moro” Medical School, 70124 Bari, Italy; ilaria.sgaramella@policlinico.ba.it (L.I.S.); alessandro.pasculli@uniba.it (A.P.); giovanna.dimeo@policlinico.ba.it (G.D.M.); angela.gurrado@uniba.it (A.G.); francesco.prete@policlinico.ba.it (F.P.P.)

**Keywords:** thyroid carcinoma, angiogenic microenvironment, prognostic factors, antiangiogenic therapy, therapeutic target, tumor microenvironment, tumor behavior, proliferation pathways, cell cycle control pathways, angiogenesis process

## Abstract

**Simple Summary:**

Angiogenesis is an essential event for the progression of solid tumors and is promoted by angiogenic cytokines released in the tumor microenvironment by neoplastic and stromal cells. Over the last 20 years, the role of the microenvironment and the implication of several angiogenic factors in tumorigenesis of solid and hematological neoplasms have been widely studied. The tumor microenvironment has also been well-defined for thyroid cancer, clarifying the importance of angiogenesis in cancer progression, spread, and metastasis. Furthermore, recent studies have evaluated the association of circulating angiogenic factors with the clinical outcomes of differentiated thyroid cancer, potentially providing noninvasive, low-cost, and safe tests that can be used in screening, diagnosis, and follow-up. In this review, we highlight the mechanisms of action of these proangiogenic factors and their different molecular pathways, as well as their applications in the treatment and prognosis of thyroid cancer.

**Abstract:**

Thyroid cancer is the most common endocrine malignancy, with a typically favorable prognosis following standard treatments, such as surgical resection and radioiodine therapy. A subset of thyroid cancers progress to refractory/metastatic disease. Understanding how the tumor microenvironment is transformed into an angiogenic microenvironment has a role of primary importance in the aggressive behavior of these neoplasms. During tumor growth and progression, angiogenesis represents a deregulated biological process, and the angiogenic switch, characterized by the formation of new vessels, induces tumor cell proliferation, local invasion, and hematogenous metastases. This evidence has propelled the scientific community’s effort to study a number of molecular pathways (proliferation, cell cycle control, and angiogenic processes), identifying mediators that may represent viable targets for new anticancer treatments. Herein, we sought to review angiogenesis in thyroid cancer and the potential role of proangiogenic cytokines for risk stratification of patients. We also present the current status of treatment of advanced differentiated, medullary, and poorly differentiated thyroid cancers with multiple tyrosine kinase inhibitors, based on the rationale of angiogenesis as a potential therapeutic target.

## 1. Introduction

Thyroid cancers are the most common endocrine malignancies and have been shown to be one of the fastest-growing malignancies worldwide over the past two decades [1,2,3]. More than 95% of thyroid carcinomas derive from follicular epithelial cells, and up to 90% of all cases are papillary thyroid cancer (PTC) [4,5]. PTCs usually grow slowly and in an indolent fashion, and their association with lymph node metastasis varies from 30% to 90% of cases [6,7]. Most thyroid carcinomas can be successfully treated with surgical resection and radiometabolic therapy, but a subset of them will progress to refractory/metastatic disease. A role of primary importance in the aggressive behavior of solid and hematological neoplasms has long been identified in the transformation of the tumor microenvironment into an angiogenic microenvironment [8,9,10]. In fact, during tumor growth and progression, angiogenesis represents a biological process uncontrolled and unlimited in time; the angiogenic switch, characterized by the formation of new vessels (i.e., the transition from the avascular to the vascular phase), induces tumor cell proliferation, local invasion, and hematogenous metastasis [8,9,11]. Identification of tumor biomarkers that might predict disease progression is a medical need. Biomarkers based on genes that appear up- or down-regulated in thyroid cancers have shown poor predictive value and cannot distinguish benign from neoplastic nodules [12]. Moreover, traditional tissue biopsies are somewhat invasive, create discomfort to the patients, and are burdened by contamination from normal tissue and sampling errors [13].

Another area where biomarkers are lacking is the identification of disease persistence after surgery/medical therapy and the ability to distinguish between complete response after treatment or recurrence of disease [13].

Angiogenesis is an essential event for the progression of solid tumors and is promoted by angiogenic cytokines released in the tumor microenvironment by tumor and stromal cells, and can also be found and measured out with a serum assay in terms of circulating angiogenic factors [14]. This is a noninvasive, inexpensive, and safe test that can be potentially used in screening, diagnosis, and follow-up of thyroid cancer patients. Here, we will review the role of angiogenesis in thyroid cancer progression, spread, and metastasis. Moreover, the potential role of proangiogenic cytokines for risk stratification of patients with thyroid cancer will be addressed, as well as the individuation of angiogenesis as a potential therapeutic target.

## 2. The Thyroid Cancer Microenvironment

Over the last 20 years, the role of the microenvironment in tumorigenesis of thyroid cancer has been well-defined [15]. The components of the thyroid cancer microenvironment (stromal cells, ST, and extracellular matrix components (ECM)) surround and support cancer cells, interacting with them by direct cell-cell and cell-extracellular matrix components interaction. Moreover, a plethora of cytokines and growth factors are produced and released in the cancer microenvironment by cancer cells as well as by ST [16]. It has been demonstrated that cancer-associated fibroblasts (CAFs) surround the tumor cells and play a role in tumor initiation and promotion, tumor cell growth, spreading, and metastasization [17]. Moreover, CAFs are involved in inflammation, metabolism, drug response, and immune surveillance [17]. There is evidence that the expression of CAF-related proteins in stromal and cancer cells varies on the basis of histologic subtype of thyroid carcinomas, BRAF^V600E^ mutation, and subtype of stroma, and an association has been reported between CAF-related proteins and poor survival [18]. It has also been demonstrated that CAFs are involved in the lymphatic spread of thyroid cancers [19], and that, in poorly differentiated thyroid cancer driven by BRAF^V600E^ mutations and loss of Pten, there is a close association between CAF infiltration and collagen I deposition in the tumor microenvironment [20]. Moreover, CAFs produce and release many angiogenic cytokines that contribute to the angiogenic process in the tumor microenvironment [21].

Tumor-associated macrophages (TAMs) are another pivotal component of the thyroid tumor microenvironment associated with tumor cell growth, spread, and poor prognosis [22,23]. In the tumor microenvironment, TAMs contribute to the anti-inflammatory status because of their high expression of interleukin-10 (IL-10) and mannose receptor (MR, CD206), and low expression of IL-12 [24,25]. It has been shown that TAMs present an M2-like activated status, differently from the M1-like circulating macrophages [26,27]. TAMs contribute to angiogenesis of tumors with an increased production of proangiogenic factors, such as vascular endothelial growth factor (VEGF), platelet derived growth factor (PDGF), and basic fibroblast growth factor (bFGF), and produce a high amount of matrix metalloproteases (MMPs), which are responsible for ECM remodeling and facilitate tumor cells spread and invasion [28,29]. The relationship between positive tryptase mast cell (MCs) infiltration and thyroid cancer invasiveness or extrathyroidal extension has been demonstrated. A significantly more abundant presence and distribution of MCs in the intratumoral and peritumoral areas of thyroid cancer has also been shown with respect to adenoma [30,31]. MCs also produce a broad spectrum of chemokines (CXCL8/IL-8, CCL25/TECK, CXCL10/IP-10, CXCL1/GRO-α), interleukins (IL-6), and other molecules (TNF-α) that are involved in the epithelial-to-mesenchymal transition (EMT) activation of thyroid cancer cells [32,33]. Moreover, MCs recruited by several tumor-derived chemotactic factors, such as stem cell factor, VEGF, chemokines, and cytokines, histologically are localized close to epithelia, fibroblasts, and blood and lymphatic vessels and are involved in wound healing, angiogenesis, and lymphangiogenesis [32,33,34]. Neutrophils are recruited by thyroid cancer cells by releasing CXCL8/IL-8 and granulocyte colony-stimulating factor [35]. In the tumor microenvironment, neutrophils produce and release angiogenic cytokines (onconstatin-M, VEGF-A) and their granule proteins (elastase), which induce cancer cell proliferation, invasiveness, and angiogenesis [36,37,38]. Our group’s complete gene expression profile study demonstrated that the microenvironment components surrounding the thyroid cancer cells express a genomic profile different from that of normal ST [39]. The results of this study indicate that interactions between tumor cells and ST induce in the ST the modulation of genes involved in the control of apoptosis, metabolism, cell movement, cell response to hypoxia, and cell proliferation [39].

### 2.1. Angiogenesis in Thyroid Cancer

Neovascularization in the cancer microenvironment is a multistep process that is necessary during the progression of solid and hematologic tumors [8,9]. It is a complex and heterogeneous process that includes three different mechanisms: (i) angiogenesis, the sprouting of newly formed vessels from mature preexisting ones; (ii) vasculogenesis, the formation of neovessels starting from endothelial precursor cells, namely hemangioblasts; and (iii) vasculogenic mimicry, the ability of tumor cells or other non-endothelial cells to complete the neovessel wall or to form a complete capillary network without vascular endothelial cells [40,41]. Moreover, tumor-related neovessel formation occurs mainly through sprouting of new capillary vessels out of preexisting ones (angiogenesis), the longitudinal splitting of existing vasculature into two functional vessels (intussusceptive angiogenesis) and the loop-shape expansion of the vessel (looping angiogenesis) involved mainly in wound healing [42,43,44]. Increased vascularity in the thyroid can occur in hyperplastic goiter, Graves’ disease, and cancer [45]. As in other solid and hematologic tumors, the microvessel density (MVD) has been shown to correlate with disease-free survival in thyroid cancers, particularly in PTC and in follicular thyroid carcinoma (FTC) [45,46,47]. Differences have been demonstrated among tumor types in the patterns of spread and metastasis, probably due to the influence of tumor metastasis route by phenotype, angiogenic or lymphangiogenic, determining a more aggressive behavior [48,49]. Thyroid adenomas, microcarcinomas, PTC, FTC, undifferentiated thyroid carcinomas, and medullary thyroid carcinomas (MTCs) present very different behaviors, clinical outcomes, and metastatic routes (lymphatic or hematic) [48,50]. These differences correlate with different angiogenic regulators released (stimulators or inhibitors), the different expression of receptors, and different extracellular matrix composition in the tumor microenvironment [50].

In thyroid tumors, angiogenesis is activated and maintained by the modulation of the genes involved in angiogenesis and response to hypoxia (HIF1A, TUFT1, BHLHB2), cell survival (RIPK5), proliferation (PTGS2, DUSP5), apoptosis (ZFP36L1, IER3), metabolism (SLCA2A3), cell organization (RAB7B) and protein degradation (SKP1, KLK-4) in the ST surrounding the tumor cells [39]. These alterations are induced and stabilized in the components of the microenvironment through the reciprocal positive and negative interactions between tumor cells and ST (endothelial cells, fibroblasts, macrophages, mast cells) and are mediated by an array of cytokines, receptors, and adhesion molecules [39,51]. Moreover, the communication between cancer cells, ST, and the various stromal cells is mediated by the release of exosomes by the thyroid tumor cells, which contribute to tumor progression, angiogenesis, and metastasis [52]. 

Evidence indicates that there is a dysregulation of miRNA in thyroid cancer that influences the hallmarks of cancer, including proliferative signaling, evading growth suppressors, resisting cell death, inducing angiogenesis, activating invasion and metastasis, and acquiring the epithelial-mesenchymal transition phenotype [53]. A role in modulating angiogenesis is played by the thyroid hormone (L-thyroxine, T4; 3,5,3′-triiodo-L-thyronine, T3) that represents a valid contributor to this process in thyroid cancers [54]. Thyroid hormone acts by binding the hormone receptor site on αvβ3 integrin and then modulating angiogenic cytokine (VEGFR and bFGFR) release via integrin activation and signaling in blood vessel cells [47,55]. This modulation is also mediated by hypoxia-inducible factor-alpha (HIF-1α), a transcription factor whose stabilization in cells is regulated by thyroid hormone via αvβ3 [56,57]. Thyroid-stimulating hormone (TSH), the glycoprotein hormone stimulating the number, size, and activity of thyrocytes as well as the synthesis of thyroid hormone, also contributes to angiogenesis stimulation [40,58,59]. TSH enhances angiogenesis and macrophage recruitment into the thyroid tumor microenvironment and then tumor cell growth through VEGF mRNA and protein induction via the protein kinase C pathway [60,61]. Iodine deprivation causes reactive oxygen species (ROS) production, stabilization of HIF-1α and VEGF release through the activation of signals in the tumor thyrocytes that induce microvascular expansion to facilitate enhanced delivery of iodide [62,63]. Iodine deficiency induces VEGF-A expression by increasing phosphorylation of ribosomal S6 kinase (p70S6K), mediated by mammalian target of rapamycin (mTOR); the latter acts as a positive regulator and AMP-activated protein kinase, in turn stimulating thyroid microvascular activation [64].

### 2.2. Angiogenic Factors

VEGF is a member of a family of six structurally related proteins, namely, VEGF-A, -B, -C, -D, -E (viral factor), and PDGF; these act by interacting with their relative receptors, which are differentially expressed in various cell types [41,65].The VEGF receptors are differentially implicated in angiogenesis stimulation (VEGF-A, -E/VEGFR-2-neuropilin (NRP)-1, -2), or lymphangiogenesis (VEGF-C, -D/VEGFR-2, -3) [41,66,67]. On the other hand, VEGF also acts on cell types different from vascular cells, modulating various biological activities and primarily tumor cell growth, spread, invasiveness, and drug resistance [68,69,70].

Our group demonstrated that, as by tumor cells, VEGF is also produced by all the cellular components of the tumor microenvironment and acts via autocrine and paracrine loops to carry out its activity [71,72]. In cancer cells and ST, VEGF expression is modulated by several pathways, including metabolic factor-induced pathways, such as hypoxia and hypoglycemia via ROS production [62,63,73]; lysophosphatidic acid (LPA), via activation of c-Jun N-terminal kinase (JNK) and nuclear factor kappa-light-chain-enhancer of activated B cells (NF-κB) [74]; PI3K/Akt signaling pathway [75]; and transcription factors, such as activator protein-1 (AP-1), NF-κB, and stimulatory protein-1 (SP-1) [76,77,78]. In thyroid carcinomas, VEGF over-expression has been correlated with increased growth, progression, invasiveness, spread, and metastasis of thyroid cancer cells [79,80,81]. A consistent increase in VEGF, VEGF-C, and angiopoietin-2 and their tyrosine kinase receptors VEGFR2, VEGFR3, and TEK receptor tyrosine kinase have been demonstrated in thyroid cancer versus normal thyroid tissues, and a strong correlation has been found between this overexpression and tumor size [50]. Moreover, the same authors showed that in the lymph node of metastatic thyroid tumors, there is an increase of VEGF-C expression and, at the same time, a reduced expression of TSP-1 near VEGF and angiopoietin-2 increased production, indicating the hematogenous metastasis capability of thyroid malignancies [50].

bFGF is an angiogenic growth factor that, by interaction with FGF receptor (high-affinity tyrosine kinase (TK) receptor) and with low-affinity heparan sulphate proteoglycans (co-receptors), induces activation, proliferation, chemotaxis, protease production, and vessel formation in endothelial cells [82,83]. In this way, it induces angiogenesis and modulates neovascularization during physiological (wound healing, inflammation) and pathological (atherosclerosis, cancer) conditions [84]. Several studies demonstrated that in thyroid tumors, the expression of bFGF and FGFR are both increased and play a role in tumor progression and angiogenesis [85,86,87,88]. As in other solid and hematologic cancers, in thyroid tumors bFGF acts as an angiogenic factor independently in the presence of other factors, such as VEGF, and directly stimulates endothelial and tumor cell growth [45].

**Figure 1:**
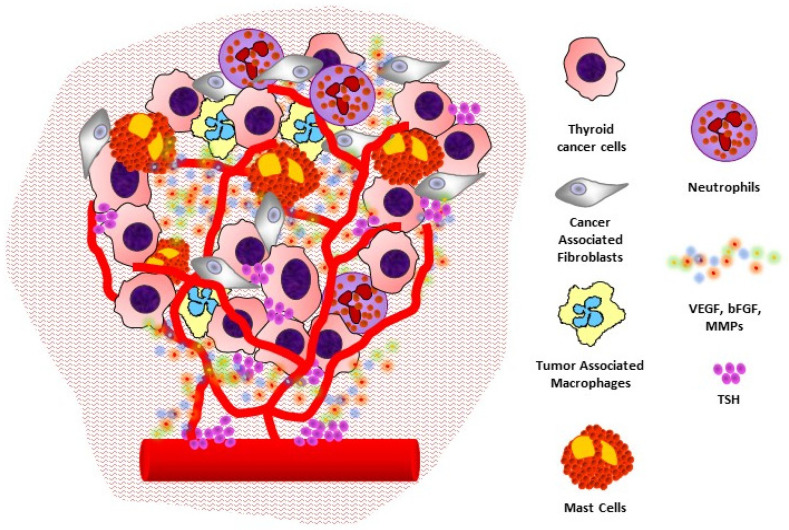
The angiogenic microenvironment of thyroid cancer: interactions between cancer cells, stromal cells, and inflammatory cells to promote angiogenesis and tumor progression.

MMPs are zinc-endopeptidases of the protease superfamily with specific proteolytic activity against a broad range of substrates located on the ECM [89,90]. MMPs are produced by thyroid tumor and microenvironment stromal cells and promote tumor growth, invasion, migration, and apoptosis inhibition. Moreover, they exert angiogenesis stimulation because the degradation of ECM causes the release of angiogenic factors stored in attachment with heparan sulphate [89,90]. The promotion of tumor growth is primarily related to MMP-2 and MMP-9 through activation of TGF-β [91]. Another growth factor, namely epidermal growth factor (EGF), is involved in promoting cell invasion and angiogenesis in thyroid carcinoma. It acts as a regulator of the production of MMP-9 through focal adhesion kinase (FAK) phosphorylation [92]. Natural inhibitors of MMPs are the tissue inhibitors of metalloproteinases (TIMPs), produced and released in the tumor microenvironment [93]. Published data from our group indicate that the MMPs’ proangiogenic and pro-tumoral activities are related to the balance of MMPs and TIMPS in the microenvironment, and that the switch toward an invasive phenotype is mainly due to increased MMP production and release, and not to the reduction of TIMPs (Figure 1) [94,95].

## 3. Angiogenesis and Prognosis

Increased MVD, lymphatic vascular density (LVD), and expression of angiogenic and lymphangiogenic factors have been demonstrated in non-neoplastic (multinodular goiter, toxic multinodular goiter, Graves’ hyperplasia) and neoplastic conditions (follicular adenoma, papillary thyroid carcinoma, incidental papillary microcarcinoma, follicular carcinoma, and medullary carcinoma) [48,96]. No clear relationship between MVD measurement and thyroid pathology has been demonstrated. In fact, de la Torre et al. showed that MVD is decreased in all thyroid diseases, and LVD is increased in papillary thyroid carcinoma and incidental papillary microcarcinoma [48]. A second study found an increased MVD in PTC compared to normal controls [96]. Other studies demonstrated a high MVD in differentiated thyroid cancers (DTCs) compared to poorly differentiated thyroid cancers and other thyroid tissue samples [97,98,99]. More consistent results have been obtained with the evaluation of VEGF. Increased distribution and intensity of VEGF-A and VEGF-C have been demonstrated in thyroid cancers compared to normal samples and autoimmune and inflammatory diseases [48,96,98,99]. However, this increased expression was not indicative of multifocal disease, distant metastases at diagnosis, or increased tumor size [48].

Examining angiogenic processes connecting the thyroid cancer cell to its microenvironment could improve many thyroid cancer management steps. The first could be identifying cytologically indeterminate nodules prone to surgical treatment, thus reducing diagnostic thyroidectomies. The second could be the efficient differentiation between aggressive and indolent DTC so that the treatment extension and approach and the follow-up modalities could be correctly adapted. An improvement to correctly identify thyroid cancer in nodules with indeterminate cytology, ruling in or out malignancy, and selecting patients for surveillance, conservative or radical surgery, was the introduction of gene classifiers to be performed on the fine needle aspiration specimen (or even micro-biopsies or surgical specimens) [100,101,102,103,104,105]. BRAF mutations, and RET-PTC, RAS and PAX8-PPARG, are the most studied factors in this field. The complete genomics of thyroid cancer subtypes will be unveiled, but caution should be exercised in the interpretation and application of the many variants and mutations that are being discovered, because many of them can also be found in benign lesions; there are also thyroid cancers that show none of the known genetic alterations [106]. BRAF mutational status, along with membranous and nuclear galectin-3, HBME-1, CK19, and estrogen receptor beta, had been associated with DTC with aggressive behavior [107]. BRAF^V600E^ mutated microcarcinomas are associated with adverse prognostic factors, whereas BRAF wild-type ones are associated with indolent behavior and a low probability of recurrence [108]. BRAF^V600E^ is associated with tall cell variant PTC, along with mutations of COL5A1, COL1A1, COL10A1, COL11A1, CCL20, and CXCL5 [109]. BRAF is the most prevalent genetic alteration in radioiodine refractory metastatic thyroid cancer patients. BRAF mutation seems to positively influence the median progression-free survival (PFS) in radioiodine refractory patients treated with tyrosine kinase inhibitor, while having a negative prognostic impact in radioiodine-sensitive PTC patients [110]. BRAF^V600E^ mutation is associated with central neck nodal metastases, but concerns were raised for its utility as a stand-alone marker in this field. Indeed, its use along with the analysis of miR-146b-3p, miR-146b-5p, and miR-222 was found to be prognostic of central neck nodal metastases preoperatively [111]. miRNA classifiers are indeed another option to identify and stratify thyroid cancer [53,112,113]. BRAF ^V600E^ appears to identify a subgroup of solitary intraglandular PTC larger than 2 cm and smaller than 4 cm, with a high risk of recurrence, for which a more aggressive treatment should be recommended [114]. There is evidence that BRAF^V600E^ reduces TSP-1 expression in anaplastic thyroid cancer, and this appears to be linked to enhanced proliferation, adherence to collagen, migration, and invasion of the neoplastic thyroid cell. Arguably, this is due to the activation of pericytes in the microenvironment of thyroid cancer, which contributes to stabilizing new vessels through the secretion of PDGRFbeta, VEGF, and other factors [115,116,117].

VEGF-C and angiopoietin-2, together with their tyrosine kinase receptors KDR, Flt-4, and TEK, were found to be increasingly expressed in thyroid cancers, especially in the transition from a prevascular to vascular phase, and this was also correlated to the tumor size, nodal invasion, and, along with a reduced expression of TSP-1, to distant metastases [50]. It is known that VEGF overexpression correlates with increased microvascular density and, similarly, a reduced expression of TSP-1 is associated with the increased microvascular count [40]. Serum VEGF levels were significantly higher in patients with metastatic thyroid cancer than in healthy subjects and patients in remission [118]. Conversely, VEGF-D serum levels were decreased in patients with metastatic thyroid cancer, and this should be linked to other factors produced by the cancer cell that inhibits the usual production from other tissues of VEGF-D [119]. Moreover, anaplastic tumors show augmented expression of VEGF [45]. The evidence of high VEGF mRNA expression and of high FAL1 expression and cyclin D1 protein levels also shows how angiogenic processes are driven along with enhanced cell proliferation in papillary thyroid cancer [120,121]. In the context of prognostic examinations for patients affected by radioiodine refractory metastatic thyroid cancer, integrin αvβ3 is essential for tumor angiogenesis, and its expression is high on the surface of activated endothelial cells in newly formed blood vessels. It has been used to trace, using 99mTc-3PRGD2 imaging, metastases that are highly neovascularized. This new angiogenesis imaging modality can provide a new tool to monitor the efficacy of antiangiogenetic therapy [122]. TSH stimulation exerts a regulatory effect on VEGF production from thyroid cancer, probably through the interaction of tissues other than the thyroid itself, and this might have prognostic and therapeutic applications, exploiting the effects of recombinant human TSH administration [60,118,123,124]. The great interest in angiogenesis for prognostic purposes in cancer appears to be tightly connected to the need for circulating markers; this is even true for thyroid cancers, thus avoiding biopsies and other procedures to stratify patients. A possible application for this aim is the assay of miRNA in exosomes, especially miR-21-5p [52,125], which is a decisive proangiogenic factor produced by thyroid cancer cells. VEGF-A and PDGF-BB have been recently indicated as potential circulating biomarkers of PTC treatment [94].

MTC lesions show overexpression of VEGF-A, VEGFR-1, and VEGFR-2 [126], but their prognostic significance is uncertain, although VEGFR-2 and EGFR seem to be related to metastasis [127]. MTC originates from embryologically different tissue, and its management is different from that of DTC. HIF-1α has been associated with an adverse prognosis for MTC [128]. HIF1-1α expression is induced by hypoxia or aberrant signaling and stimulates the expression of VEGF and angiogenesis.

Moreover, MTC shows PSMA expression in the neovasculature, and microvessels showing positivity for PSMA are prognostically favorable. PSMA may become a target for imaging and peptide radioligand therapy [129]. It has been shown that collagen deposition and cross-linking and fibroblast presence in the thyroid cancer microenvironment play a prognostic role, indicating an advanced or aggressive disease, and are driven by PTEN loss BRAF^V600E^ [20,130,131,132,133]. The overexpression of MMPs leads to increased VEGF and FGF secretion, related to tumor growth and invasiveness [134,135]. The ratio of MMP-2 to TIMP-2 expression is a prognostic factor for surgically treated MTC, and both of these metalloproteinases play a role in PTC [136,137,138]. MMP-9 expression is upregulated in PTC and might be a prognostic indicator for more advanced-stage cancer [139,140,141,142]. The prognostic role of other components of the extracellular matrix of thyroid cancer tangentially involved in angiogenetic processes, such as macrophages and T lymphocytes, is yet to be clarified [15].

## 4. Preclinical and Clinical Evidence for Antiangiogenic Therapy

Therapeutic options for thyroid cancers are variable following clinical-pathological staging. Localized and low-risk DTC benefit from surgical treatment (total thyroidectomy) followed by thyroid hormone suppression and adjuvant thyroid ablative therapy with radioactive iodine (RAI) treatment. This management could be applied to 85% of papillary and follicular subtypes [143,144].

In metastatic disease, patients are usually managed with a combination of surgery and RAI treatment. The prognosis depends on metastasis location (if it is suitable for a complete surgical resection) and post-operative radioiodine uptake in the tumor tissue. In the same setting of total thyroidectomy, indeed, patients could be submitted to therapeutic central neck dissection or lateral neck dissection in case of preoperative or intraoperative inspection of lymph nodes that are deemed to be pathologic [145]. However, some tumors are refractory to T4-mediated TSH suppression as well as RAI therapy. Approximately two-thirds of these patients may develop distant metastases resulting in much poorer overall survival rate and a poor prognosis [143,144].

In recent years, the scientific community’s effort has been focused on the study of several molecular pathways involved in cancer development. Proliferation pathways, cell cycle control pathways, and angiogenesis processes have been largely evaluated, allowing the identification of mediators that may be useful targets for new anticancer treatments [146]. Multiple tyrosine kinase inhibitors (TKIs) have been identified, and their efficacy on different molecular pathways has been the primary end point of several studies in search of further treatment options for recurrent/metastatic thyroid cancer.

Vandetanib was the first TKI approved by the Food and Drug Administration (FDA) for the treatment of patients with symptomatic, unresectable, locally advanced, or metastatic MTC in the USA (2011) and Europe (2013). This drug acts on EGF, RET, VEGF, and VEGF receptors, leading to a downregulation of proliferative, angiogenic pathways and mediation on apoptosis pathways. The effects have been demonstrated in two phase 2 clinical trials and one phase 3 clinical trial (ZETA trial) in which patients with advanced unresectable MTC were blindly randomized to receive vandetanib at 300 mg daily or placebo [147].

This study demonstrated a significantly longer median PFS duration compared to the placebo group, with a partial response in 44% of cases. A significant difference in the objective response rates and disease control rates as well as in the biochemical response was also discussed. Moreover, vandetanib has also been tested in RAI-refractory DTC patients. A randomized, double-blinded, placebo-controlled phase 2 clinical trial evaluated the effect of this TKI, showing a statistically significant increase in the PFS of patients treated with vandetanib compared to the placebo group [143,148,149].

The second TKI, approved by the FDA in 2012 for the management of advanced and symptomatic MTC, was cabozantinib. This is a TKI with function on the hepatocyte growth factor receptor, RET, and VEGF2 receptors, leading to downregulation or inhibition of angiogenic, proliferative, and apoptotic pathways.

A phase 3 study (EXAM study) evaluated the effect of cabozantinib, showing a statistically significant longer median of PFS in patients treated with the drug (140 mg per day) with respect to those treated with placebo (11.2 versus 4.0 months). In this study, cabozantinib was associated with significant but manageable toxicity [143,149,150].

Sherman et al. published the results of an exploratory analysis of phase 3 trial data evaluating the influence of rearranged during transfection (RET) and RAS (HRAS, KRAS, and NRAS) mutations on cabozantinib clinical activity. They concluded that cabozantinib provides the most significant clinical benefit to patients with MTC who have RET M918T or RAS mutations [151].

Comparing vandetanib with cabozantinib, it seems that vandetanib is more tolerable than cabozantinib, making it the drug of choice for fragile and older patients. This is probably due to differences in patient selection for the two studies in phase 3. In the ZETA study, non-progressive but symptomatic patients were also studied, while the EXAM study enrolled only progressive cases, with adverse events due to cabozantinib higher than the adverse events observed in the ZETA trial. Cabozantinib has stronger antiangiogenic effects than vandetanib; therefore it could be strongly considered in cases of rapid MTC progression.

Cabozantinib and vandetanib have been tested on DTC, without approval [143,149].

In November 2013, sorafenib was the first multiple TKI to be approved by the FDA for the treatment of progressive metastatic DTC refractory to RAI treatment.

It has a documented efficacy in inhibiting all RAF kinases with a specific function on VEGF receptors 1–3, PDGFRB, and RET, which confers to sorafenib proapoptotic propriety and angiogenic effect fitting a molecular rationale for the treatment of all histological subtypes of thyroid cancer. Indeed, some studies have analyzed the use of Sorafenib in patients with metastatic thyroid cancer not suitable for curative surgery, RAI, or radiotherapy with a dose of 400 mg twice a day; these studies report partial response in 32% of cases, stable disease beyond six months, and a toxicity profile similar to that observed in previous studies and managed with dose delay or reduction [152]. Brose et al. investigated sorafenib in a multicenter, randomized, double-blind, placebo-controlled, phase 3 trial (DECISION). Sorafenib was used with the same administration in patients with RAI-refractory, locally advanced, or metastatic DTC that had progressed within the previous 14 months. The results showed how significantly sorafenib improved PFS compared to placebo [153].

Another TKI, lenvatinib, was approved in February 2015 in many countries to treat advanced DTC refractory to RAI. This is a multitargeted TKI of the VEGFRs 1, 2, and 3, FGFRs 1 through 4, PDGFR α, RET, and KIT signaling networks. In a phase 1 study, lenvatinib was associated with a partial response in thyroid cancer as well as melanoma, endometrial, and renal cancers. Subsequently, Sherman et al. performed a phase 2 study involving patients with DTC refractory to RAI, analyzing clinical activity and efficacy of levantinib [154]. Further, a phase 3, randomized, double-blind, multicenter study involving patients with progressive thyroid cancer refractory to iodine-131 was performed (SELECT trial). Schlumberger et al. randomized 261 patients to receive lenvatinib (at a daily dose of 24 mg per day in 28-day cycles) and 131 patients to receive placebo, showing PFS benefit and a high response rate associated with lenvatinib compared to placebo [155]. In an exploratory analysis, Wirth et al. examined treatment-emergent hypertension and its relationship with lenvatinib efficacy and safety in SELECT. They concluded that TE-HTN was significantly correlated with improved outcomes in patients with radioiodine-refractory DTC, indicating that hypertension may be predictive of lenvatinib efficacy in this population [156].

Based on these studies, sorafenib and lenvatinib are now recommended by the National Comprehensive Cancer Network to treat progressive, RAI-refractory DTC [157].

A multicenter phase II study investigated the efficacy and safety of dovitinib in advanced or RAI refractory thyroid cancer. This is another oral TKI with a documented activity on VEGFR, PDGFR, and RET pathways and a unique feature of inhibiting FGFRs. After a phase I study, in which the antitumor activity of dovitinib was evaluated on metastatic renal cell carcinoma [158], Indeed, Lim et al. tested dovitinib orally 500 mg once daily for five consecutive days followed by a 2-day rest every week in patients with PTC, FTC, and MTC with refractory disease. The study showed that dovitinib has modest activity with manageable toxicity with an overall response rate of 20.5% and a disease control rate of 69.1% compared to a relatively short PFS [144].

Another important multitarget tyrosine kinase inhibitor, anlotinib, has been tested for advanced refractory solid tumors. It has a referred action on tumor angiogenesis and growth with a proved effective target on VEGF and their receptors with an inhibition capacity 500 times stronger than sorafenib. Anlotinib also acts on tumor progression and cell proliferation by inhibiting PDGFR alfa/beta, c-Kit, Ret, Aurora-B, c-FMS, and discoidin domain receptor 1 and carrying mutations in PDGFR alfa, c-Kit, Met, and EGFR. In a phase I, open-label study on patients with various types of solid tumor including MTC, Sun et al. showed that at a dose of 12 mg once daily on the 2/1 schedule, anlotinib had a toxicity profile in agreement with that reported for sorafenib and a substantial, broad-spectrum antitumor potential [146]. Subsequently, in a phase II clinical trial, the antitumor activity of anlotinib in patients with advanced or metastatic MTC was confirmed, also demonstrating a manageable adverse event profile [159].

A TKI with selective action on VEGFR1-3, axitinib, has been tested in patients with different types of thyroid cancers. Initially approved for the treatment of renal cell carcinoma, it has been tested in two phase 2 clinical trials for thyroid cancers with a recommended dose of 5 mg twice daily. Both studies described a clinical benefit in refractory and progressive thyroid cancer, tolerability, and a safety profile for axitinib as first-line therapy [160,161].

Pazopanib is another antiangiogenic TKI acting on VEGFR1-3, FGF1/2, PDGF, KIT, and RET receptors. After being approved for renal cancers, it has been tested (at a dose of 800 mg daily) in a phase II clinical trial in patients with metastatic, rapidly progressive, radioiodine-refractory DTC. Another phase II clinical trial tested the antitumor activity on MTC and anaplastic thyroid cancer [162,163,164].

Recently, another phase II clinical trial of pazopanib in RAI refractory DTC patients with progressive disease confirmed the clinical activity and manageable toxicities of the drug, examining in parallel biomarkers that might precede therapeutic response. However, no predictive biomarkers were found to facilitate a robust early identification of patients likely to respond to pazopanib therapy [165].

Sunitinib is an antiangiogenic TKI able to inhibit the VEGF1–3, PDGF, KIT, and RET receptors. As with other TKIs, it was first approved for other cancers such as renal cell carcinoma and pancreatic neuroendocrine carcinoma and then analyzed for RAI refractory DTC and MTC with FDG-PET-avid disease. Three phase 2 clinical trials reported on sunitinib administered at 37.5 mg daily on a continuous basis. All studies concluded that sunitinib exhibits significant anti-tumor activity in patients with advanced DTC and MTC, with some common adverse events such as fatigue, diarrhea, hand-foot syndrome, neutropenia, and hypertension [166,167,168].

The knowledge of thyroid tumor behavior, with its ability to produce cytokines and chemokines and consequently promote tumorigenesis, has guided the scientific community for many years in identifying the best TKI. This has led to the testing and approval of different therapeutic options for advanced thyroid tumors that previously had no possibility of treatment (Table 1).

Recently, the development and approval of immunotherapeutics for cancer and the identification of immune checkpoint inhibitors have modified the treatment landscape for many malignancies, taking advantage of the capacity of restoring the state of immunosurveillance on some tumors that were able to evade it [169,170].

Starting from the evidence of increased frequency of aggressive regulatory T cells and the correlation between expression of PD-1 ligand and the worse prognosis for recurrent PTC, the immune system components have been largely studied as therapeutic targets useful in the treatment of thyroid cancer. These are the new fields to be explored in the analysis of target therapy [169].

## 5. Conclusions

The study of several molecular pathways and the tumor microenvironment involved in cancer development has recently guided the scientific community’s effort to clarify tumor behavior. Indeed, proliferation pathways, cell cycle control pathways, and the processes of angiogenesis have been largely evaluated, providing new tools useful in screening, diagnosis, and follow-up of thyroid cancer and allowing the identification of mediators that may be potential targets for new anticancer treatments.

VEGF and its receptors appear to be the major players in the angiogenesis process of thyroid tumors. There have been numerous efforts toward understanding the signaling mechanisms driven by BRAF^V600E^ mutation and loss of Pten as a contribution to the angiogenic process in the thyroid tumor microenvironment. For these reasons, antiangiogenic therapy is used in all histological subtypes of thyroid cancer. In addition, to date, a number of inhibitors of the RAS, RAF and MEK pathways and other types of molecular target therapy have been shown to be effective in vitro and require clinical confirmation.

The tumor microenvironment presents numerous barriers that prevent access to chemotherapies, not only rendering them often ineffective but also potentially increasing the tumor cells’ aggressiveness.

Prognostic indicators are based on dynamic interactions between multiple types of cells, especially those with immune functions belonging to the tumor microenvironment.

Further studies are needed to increase knowledge of the tumor microenvironment and to evaluate its changes and remodeling as thyroid cancer progresses.

Additionally, other studies should aim to determine the role of the immune system in thyroid cancer.

## Figures and Tables

**Table 1:** Drugs studied in treatment of thyroid cancer: Tyrosine kinase inhibitors and their multitarget activity.

Drugs	Mechanism of Action: Target(s)	Dose	Study Phase	Tested or Approved for Thyroid Cancer Treatment
Vandetanib	-EGF-R	300 mg daily	Phase III clinical trial (ZETA trial) [147]	Treatment of patients with symptomatic, unresectable, locally advanced or metastatic MTC
	-RET			
	-VEGF-R			
Cabozantinib	-MET	140 mg per day	Phase III clinical trial (EXAM trial) [150]	Management of advanced and symptomatic MTC
	-RET			
	-C-Kit			
	-VEGF-R2			
Sorafenib	-BRAF	400 mg twice a day	Phase III clinical trial (DECISION trial) [153]	Treatment of progressive metastatic DTC refractory to RAI treatment.
	-VEGF-R1			
	-VEGF-R3			
	-PDGF-R			
	-C-Kit			
	-RET			
Lenvatinib	-VEGF-R1	24 mg once daily	Phase III clinical trial (SELECT trial) [155]	Treatment of advanced DTC refractory to RAI
	-VEGF-R2			
	-VEGF-R3			
	-FGF-R1			
	-FGF-R4			
	-PDGF-Rα			
	-RET			
	-C-Kit			
Dovitinib	-VEGF-R	500 mg once daily for five consecutive days followed by a 2-day rest every week	Phase II clinical trial [144]	Treatment of advanced or RAI refractory thyroid cancer (PTC, FTC, and MTC)
	-PDGF-R			
	-RET			
	-FGF-R			
Anlotinib	-PDGF-R α/β	12 mg once daily	Phase II clinical trial [159]	Treatment of advanced refractory solid tumors (including MTC)
	-C-Kit			
	-RET			
	-AURORA-B			
	-C-FMS			
	-DDR1 (DISCOIDIN DOMAIN RECEPTOR 1)			
	-MET			
	-EGF-R			
Axitinib	-VEGF-R1	5 mg twice daily	Phase II clinical trial [161]	Treatment of different types of thyroid cancers
	-VEGF-R3			
Pazopanib	-VEGF-R1	800 mg daily	Phase II clinical trials [162,163,164]	Treatment of different types of thyroid cancers (RAI refractory DTC, MTC, and anaplastic thyroid cancer)
	-VEGF-R3			
	-FGF-R1/2			
	-PDGF-R			
	-C-Kit			
	-RET			
Sunitinib	-VEGF-R1	37.5 mg daily	Phase II clinical trials [166,167,168]	Treatment of RAI refractory DTC and MTC with FDG-PET-avid disease
	-VEGF-R3			
	-PDGF-R			
	-C-Kit			
	-RET			
